# 小细胞肺癌骨髓转移患者的临床分析

**DOI:** 10.3779/j.issn.1009-3419.2018.05.08

**Published:** 2018-05-20

**Authors:** 轶群 车, 扬 罗, 迪 王, 迪 沈, 琳 杨

**Affiliations:** 1 100021 北京，国家癌症中心/中国医学科学院北京协和医学院肿瘤医院检验科 Department of Clinical Laboratory, National Cancer Center/Cancer Hospital, Chinese Academy of Medical Sciences and Peking Union Medical College, Beijing 100021, China; 2 100021 北京，国家癌症中心/中国医学科学院北京协和医学院肿瘤医院内科 Department of Medical oncology, National Cancer Center/Cancer Hospital, Chinese Academy of Medical Sciences and Peking Union Medical College, Beijing 100021, China; 3 北京，国家癌症中心/中国医学科学院北京协和医学院肿瘤医院病理科 Department of Pathology, National Cancer Center/Cancer Hospital, Chinese Academy of Medical Sciences and Peking Union Medical College, Beijing 100021, China

**Keywords:** 肺肿瘤, 小细胞癌, 骨髓转移, 预后, Lung neoplasmas, Small cell cancer, Bone marrow metastases, Prognosis

## Abstract

**背景与目的:**

小细胞肺癌（small cell lung cancer, SCLC）恶性程度高，早期易发生骨髓转移，但其相关报道有限。本研究分析SCLC骨髓转移患者的临床表现，实验室检查、治疗以及预后。

**方法:**

回顾性分析26例SCLC骨髓转移患者的临床资料，分析其预后相关因素。

**结果:**

26例SCLC骨髓转移患者的中位年龄57岁，从诊断SCLC到确诊骨髓转移的中位时间为8天。多数患者（96.2%）同时伴有其他脏器转移。最常见的实验室异常为乳酸脱氢酶增高19例（73.1%），血小板减少和碱性磷酸酶增高各11例（42.3%），贫血7例（26.9%）。全组在诊断骨髓转移后，6例未化疗，20例化疗，其中16例至少接受了2个周期化疗。全组患者确诊骨髓转移后的中位生存期为15.7周（0.1周-82.9周），接受化疗患者的生存期显著优于未化疗者（*χ*^2^=33.768, *P* < 0.001）。多因素分析显示未化疗是独立的预后不良的因素（*P* < 0.05）。

**结论:**

SCLC骨髓转移患者的生存期短，化疗可延长患者的生存。

## Abstract (en)

**Background and objective:**

Small cell lung cancer (SCLC) is highly malignant and prone to bone marrow metastasis in early stage, but its related reports are limited. This study analyzed the clinical feature, laboratory examination, treatment and prognosis of SCLC patients with bone marrow metastasis.

**Methods:**

The clinical data of 26 SCLC patients with bone marrow metastasis were analyzed retrospectively. Prognostic factors were evaluated.

**Results:**

The median age of 26 patients was 57 years and the median time from diagnosis of SCLC to confirmed bone marrow metastases was 8 d. Most patients (96.2%) were accompanied by other organ metastases. The most common laboratory abnormalities were elevated lactate dehydrogenase in 19 cases (73.1%), thrombocytopenia and elevated alkaline phosphatase respectively in 11 cases (42.3%) and anemia in 7 cases (26.9%). Twenty patients had received chemotherapy and the remaining 6 patients had not. Of this group, 16 patients received at least 2 cycles of chemotherapy after the diagnosis of bone marrow metastasis. The median survival time was 15.7 wk (0.1 wk-82.9 wk) after diagnosis of bone marrow metastasis. The survival of patients with chemotherapy was significantly better than that of those without chemotherapy (*χ*^2^=33.768, *P* < 0.001). Multivariate analysis showed that no chemotherapy was independent poor prognostic factors (*P* < 0.05).

**Conclusion:**

The SCLC patients with bone marrow metastasis have short survival, whereas chemotherapy can extend the survival of patients.

肺癌是中国癌症相关死亡的主要原因^[1]^。与非小细胞肺癌相比，小细胞肺癌（small cell lung cancer, SCLC）恶性程度高，具有早期播散的倾向，对化疗的敏感性高。约2/3的SCLC患者在初次就诊时已经是广泛期，存在脑、肝、肾上腺、骨及骨髓等远处转移，以足叶乙甙为基础的化疗是广泛期SCLC最主要的的治疗手段，骨髓抑制是化疗的剂量限制性毒性，而骨髓转移可导致患者的造血功能下降，严重影响患者对化疗的耐受性，进而导致SCLC骨髓转移患者的生存期短^[2]^。因此，研究SCLC骨髓转移的早期征象对患者的治疗和预后至关重要。本研究现分析26例SCLC骨髓转移患者的相关资料，以期对其临床典型征象和治疗策略进行初步探讨。

## 1 资料与方法

### 1.1 一般资料

收集中国医学科学院肿瘤医院2010年1月-2016年1月收治的26例SCLC骨髓转移患者的资料。所有患者均经病理细胞学证实为SCLC，来源主要包括支气管镜活检标本（14例，60.7%）、转移淋巴结穿刺活检标本（6例，26.1%）、痰细胞学标本（2例，8.7%）和肺穿刺活检标本（1例，4.3%）。临床分期检查主要包括体格检查、血液学检查、胸部计算机断层扫描（computed tomography, CT）或正电子发射计算机断层显像（positron emission tomography CT, PET-CT）、颈部及腹部超声、脑核磁和骨扫描等，分期采用VALSG（Veterans Administration Lung Study Group）两分期法（局限期：局限于一侧胸腔，伴或不伴区域淋巴结转移，可包括于一个放射野范围内的病变；广泛期：超出一侧胸腔，包括胸腔积液和心包积液或其他远处转移的病变）。诊断骨髓转移和原发性疾病的时间间隔 < 1个月定义为骨髓转移和SCLC同时发生。所有患者的骨髓转移均有骨髓穿刺细胞学证实，由两名专家检查所有骨髓涂片并分别进行评估。本研究获得中国医学科学院肿瘤医院伦理委员会批准。

### 1.2 方法

收集所有患者的实验室检查，包括血常规，肝肾功能，凝血功能，碱性磷酸酶（alkaline phosphatase, ALP）和乳酸脱氢酶（lactate dehydrogenase, LDH）水平，SCLC相关肿瘤标志物神经元特异性烯醇化酶（neuron-specific enolase, NSE），白细胞减少，血小板减少症和贫血分别定义为白细胞总数（white blood cell, WBC） < 4.0 G/L，血小板 < 100 G/L和Hb < 110 g/L。最后随访截至日期为2017年8月30日。

患者确诊骨髓转移后，6例未化疗（2例拒绝化疗，2例肝功能显著异常，1例出血倾向，1例合并感染），14例接受足叶乙甙联合铂类方案（顺铂9例，卡铂5例），6例接受不含足叶乙甙方案（4例环磷酰胺+蒽环类+长春新碱，1例紫杉醇+异环磷酰胺，1例伊立替康+奈达铂），20例化疗患者中，有4例患者因血象持续下降或胆红素升高只接受1个周期的化疗。

### 1.3 统计学方法

采用SPSS 22.0统计软件进行数据分析，采用*Kaplan-Meier*法进行生存分析，*Log-rank*法行单因素预后分析，*Cox*风险回归模型进行多因素分析。*P* < 0.05为差异有统计学意义。

## 2 结果

### 2.1 患者的一般情况

26例患者的中位年龄57岁（49岁-78岁），男性21例（80.8%），女性5例（19.2%）。美国东部肿瘤协作组（Eastern Cooperative Oncology Group, ECOG）评分0分2例（7.7%），ECOG评分1分15例（57.7%），ECOG评分2分9例（34.6%）。22例（84.6%）患者初诊时即为广泛期，其中19例存在骨髓转移，其他3例分别在病程的2个月、9个月和10个月诊断骨髓转移。4例初诊为局限期的患者分别在病程的4个月、8个月、9个月和14个月诊断骨髓转移，从诊断SCLC到确诊骨髓转移的中位时间为8天（-3天-408天）。本组患者发生骨髓转移时均伴有淋巴结转移，其他合并的脏器和/或软组织转移依次为肝（16/26）、骨（15/26）、肺（7/26）、肾上腺（5/26）、脑（5/26）、胰腺（3/26）、胸膜（3/26）、皮下（1/26）、肾（1/26），只有1例患者不合并其他脏器和/或软组织转移，9例患者的脏器和软组织转移部位数目≥3个。

### 2.2 发现骨髓转移时的临床表现

26例患者中，13例无特殊临床征象，常规进行骨髓穿刺检查确诊骨髓转移，10例因为外周血异常，3例因化疗或放疗后血小板下降明显且恢复缓慢接受骨髓穿刺。只有1例伴发出现黏膜出血，部分患者诉乏力、咯血和骨痛，但因和SCLC原发病以及骨转移的症状重叠，对提示骨髓转移无临床意义。

### 2.3 SCLC骨髓转移患者的血液学指标分析

26例患者中，12例（46.2%）患者血常规正常，1例（3.8%）患者三系下降，5例（19.2%）患者贫血和血小板二系下降，单纯血小板、单纯WBC下降和单纯贫血下降分别为5例（19.2%）、2例（11.5%）和1例（7.7%）。7例贫血患者均表现为正细胞正色素性。生化检查显示19例（73.1%）LDH升高，中位数和四分位数间距分别为447.5 U/L和612.0 U/L（range: 148.0 U/L-1, 258.0 U/L）；11例（42.3%）患者ALP升高，其中位数和四分位数间距分别为102.0 U/L和134.0 U/L（range: 53.0 U/L-414.0 U/L）。所有患者的肺肿瘤标志物NSE均有不同程度的升高，中位数及四分位间距分别为98.36 ng/mL和334.75 ng/mL（range: 5.24 ng/mL-2094 ng/mL）。

### 2.4 SCLC骨髓转移患者的骨髓像和外周血涂片

骨髓涂片检查显示骨髓增生活跃，均可见成团及散在分布的癌细胞，转移癌细胞具有胞体大，胞核大、可见多核，胞质、胞核、核仁深染等特点。转移癌细胞形态与原发灶细胞类似，常成簇，成团互相挤压或融汇在一起（图 1）。外周血涂片中，5例可见幼粒和幼红细胞，5例只见到幼粒细胞，1例只见到幼红细胞，15例无幼稚细胞（图 2）。

**1:**
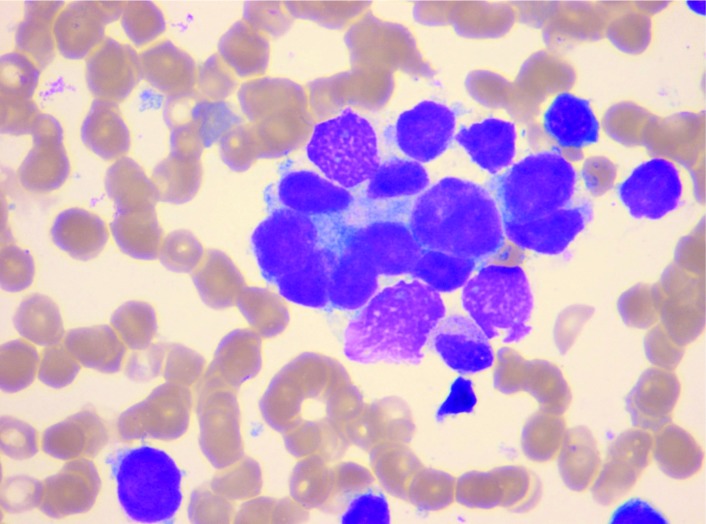
骨髓的转移癌细胞 Metastatic cancer cells clustered in bone marrow

**2:**
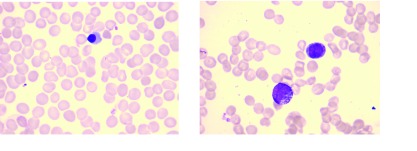
外周血涂片可见幼红和幼粒细胞 Erythroblasts and granulocytes in peripheral blood

### 2.5 SCLC骨髓转移患者的治疗和生存的关系

全组患者确诊骨髓转移后的中位生存期为15.7周（0.1周-82.9周），诊断骨髓转移后接受化疗的患者的生存期显著优于未化疗的患者（*χ*^2^=33.768, *P* < 0.001），见图 3。我们将4例因不能耐受毒副反应仅接受1周期化疗的患者归为未治疗，结果显示2周期化疗后评价肿瘤缓解的患者的生存期显著优于未缓解（疗效稳定和进展）或未治疗的患者（*χ*^2^=14.726, *P*=0.001）。

**3:**
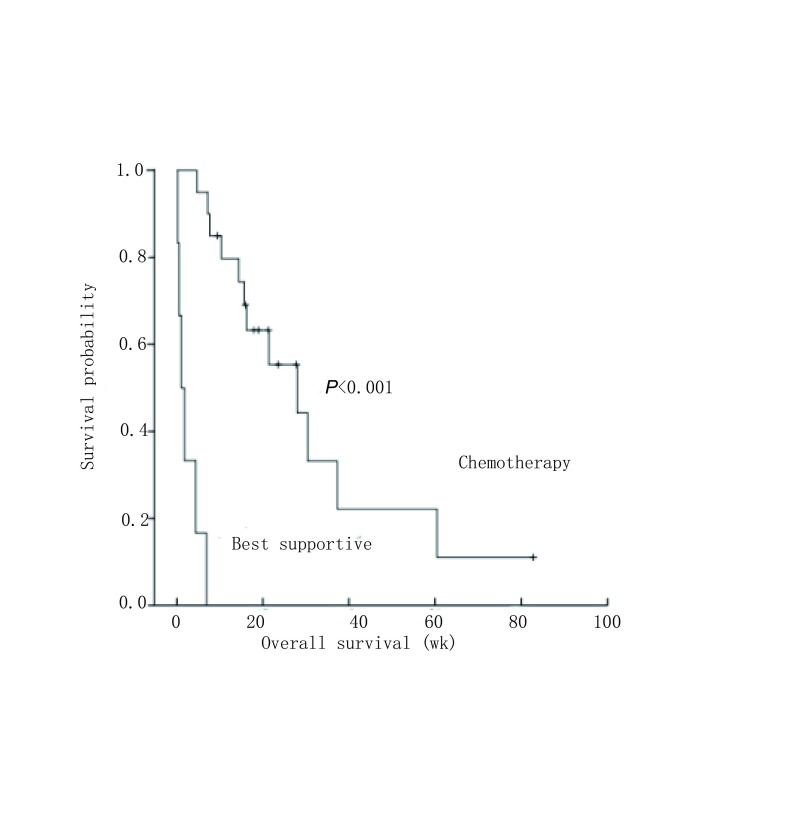
SCLC骨髓转移患者的生存曲线 Survival analysis of SCLC patients with bone marrow metastases

### 2.6 SCLC骨髓转移患者的预后

对患者的年龄（< 60岁或≥60岁）、性别、ECOG评分（< 2或≥2）、同时或异时骨髓转移、合并脏器转移数目（< 3个或≥3个），血小板 < 75 G/L或≥75 G/L，LDH（正常或升高），治疗（化疗或未化疗）进行单因素分析结果显示，血小板 < 75 G/L，LDH升高，未化疗患者的生存显著缩短（*P* < 0.05）（表 1）。将单因素分析中*P* < 0.05的3个因素进行多因素回归分析结果显示：只有未化疗是独立的预后不良的因素（*P* < 0.05）（表 2）。

**1:** SCLC骨髓转移患者的单因素预后分析 Single-factor prognostic analysis of SCLC patients with bone marrow metastases

Clinical factors	*n*	Median survival (mon)	*χ*^2^	*P*
Gender			3.346	0.067
Male	21	15.57		
Female	5	37.29		
Age (yr)			1.444	0.229
< 60	20	21.43		
≥60	6	4.29		
ECOG score			0.674	0.412
< 2	17	21.43		
≥2	9	4.57		
Bone marrow metastasis			2.331	0.127
Simultaneous	20	28.00		
Metachronous	6	7.14		
Number of organs metastasis			< 0.001	0.985
< 3	17	14.29		
≥3	9	21.43		
PLT			8.700	0.003
< 75 G/L	10	4.29		
≥75 G/L	16	28.00		
LDH			5.339	0.021
Normal	7	60.43		
Increased	19	14.29		
Chemotherapy			33.768	< 0.001
Yes	20	28.00		
No	6	1.00		
PLT: blood platelet; LDH: lactate dehydrogenase; ECOG: Eastern Cooperative Oncology Group; SCLC: small cell lung cancer.				

**2:** 预后因素的*Cox*回归分析 Multivariate prognosis analysis by *Cox* model

Factors	B	df	*P*	Exp(B)	95%CI for Exp(B)	
					Lower	Upper
PLT < 75 G/L	-0.246	1	0.742	0.782	0.180	3.385
Increased LDH	1.221	1	0.132	3.392	0.692	16.630
No chemotherapy	-3.521	1	0.004	0.030	0.003	0.335

## 3 讨论

骨髓转移癌是指原发于髓外组织或器官的恶性肿瘤细胞经血行或淋巴道转移至骨髓而引起的临床及血液学改变的一组疾病，多发生于乳腺癌、前列腺癌、肺癌和胃癌等恶性肿瘤^[3]^，目前主要依靠骨髓抽吸和骨髓活检诊断。由于骨髓转移干扰患者的造血功能，降低患者对化疗的耐受性，影响患者的预后，所以检测实体瘤骨髓转移，对制定恰当的治疗方案和剂量以及判断预后等方面具有重要的临床意义。然而，由于原发肿瘤不同，肿瘤的位置和相应的血液或淋巴引流不同，骨髓受累程度不同，导致了骨髓转移的临床表现复杂多样，尤其是在骨髓转移的早期阶段，常因缺乏临床典型症状而容易被临床医生忽略^[4]^。

SCLC生长迅速，多数患者初诊时就已经存在远处转移，如肺、肝、骨、脑、肾上腺和骨髓等部位。Zych^[5]^对146例SCLC进行双侧骨髓穿刺细胞学检查，结果发现28例（19.2%）患者存在骨髓转移。由于骨髓抽吸和/或骨髓活检是损伤性操作，部分患者出于各种原因拒绝接受，故SCLC骨髓转移的发生率存在被低估。本研究发现只有1例患者伴发出现黏膜出血，部分患者诉乏力、咯血和骨痛，但因和SCLC原发病以及骨转移的症状相重叠，临床表现对提示骨髓转移意义不大。最常见的实验室异常为LDH增高19例（73.1%），LDH是细胞进行糖酵解过程中的一种重要的氧化还原酶，恶性肿瘤组织的糖酵解高于正常组织，尤其是恶性程度高、分期晚的患者LDH升高明显，且预后差^[6]^，因此，伴有LDH增高的SCLC患者应该接受骨髓穿刺检查。本组患者接受骨穿检查的原因包括：10例因为外周血异常以及3例因化疗或放疗后后血小板下降明显且恢复缓慢，13例患者的血常规正常而因病变广泛常规进行了骨髓穿刺，并且19例患者在初诊时即存在骨髓转移，说明SCLC可以发生早期骨髓转移，也可以出现在病程中，可以引发外周血常规改变，但是血常规正常不能排除骨髓转移，而当血常规尤其是血小板下降时，临床医生经常选择减量化疗或者当血小板低于50 G/L时，多数医生会停止化疗，从而影响患者的生存期，所以我们建议对广泛期患者常规进行骨髓穿刺，局限期的患者，虽然单纯骨髓转移的可能性小于5%，但当伴随LDH和/或外周血常规异常时，需要进行骨髓穿刺，必要时双侧骨髓穿刺，明确骨髓情况。

此外，由于肿瘤细胞浸润骨髓，破坏骨髓屏障，所以患者的外周血涂片可出现幼稚细胞^[7]^，本研究26例患者中，11例（42.3%）患者的外周血涂片中可以看到幼粒和/或幼红细胞，如果患者不配合骨穿或因为PLT显著下降，不适宜进行骨穿时，可以先行外周血涂片，有助于临床诊断。

SCLC骨髓转移患者虽然对化疗的耐受性下降，但对化疗仍然敏感，Asai等^[8]^曾报道了1例低剂量氨柔比星治疗SCLC骨髓转移达完全缓解的病例。罗志国^[2]^报道含足叶乙甙的化疗方案可以延长SCLC骨髓转移患者的生存。本研究发现相比于未化疗的患者，化疗可以显著延长SCLC骨髓转移患者的生存期，虽然。不同的化疗方案对患者的生存没有影响，但是和治疗无效或未治疗的患者相比，化疗有效者的生存期显著延长。本研究对多个因素进行了预后分析，发现年龄、性别、ECOG评分、同时或异时骨髓转移、合并脏器转移数目对患者的预后均无影响，血小板＜75 G/L，LDH升高，未化疗患者的生存显著缩短（*P* < 0.05），但只有未化疗是独立的预后不良因素（*P* < 0.05）。

综上所述，临床医生应当熟悉SCLC骨髓转移的临床特点和实验室检查，SCLC的恶性程度高，容易早期发生骨髓转移，应积极进行骨髓穿刺进行诊断，尤其是广泛期和伴有血常规异常以及LDH升高的患者，发生骨髓转移后，应积极给予有效的化疗以延长SCLC患者的生存期。
